# A Survey of Primary Care Administrators’ Experiences During Integration of Pharmacists into Team-Based Primary Care in British Columbia

**DOI:** 10.1177/08404704251388321

**Published:** 2025-11-10

**Authors:** Peter J. Zed, Arwa Nemir, Peter S. Loewen, Anita I. Kapanen, Anupama Salil

**Affiliations:** 1 Faculties of Pharmaceutical Sciences and Emergency Medicine, 8166 University of British Columbia, Vancouver, British Columbia, Canada

## Abstract

Team-based primary care involving a pharmacist provides many benefits, including improved patient outcomes, increased efficiency, and enhanced patient and clinician satisfaction. This article reports findings from a provincial survey to describe primary care network administrators’ experiences during integration of pharmacists into interprofessional team in primary care networks, and to describe the facilitators and barriers of team-based care. Health service administrators reported an overall high degree of satisfaction with their experiences collaborating with the program team and with pharmacists in a developing team-based primary care model. Barriers to team-based care included lack of integrated electronic medical record preventing efficient access and sharing of patient information/communication, lack of role clarity and scope of practice among team members, and lack of co-location precluding relationship building, timely care, and optimization of the team. These barriers should be addressed to optimize the effectiveness and efficiency of the team as the model of primary care continues to evolve.

## Introduction

Interprofessional primary care provides many benefits, including improved patient outcomes, increased team efficiency, and enhanced patient and clinician satisfaction.^1-3^ Benefits to patients who receive care from a team of healthcare providers that includes a pharmacist include identification and resolution of Drug Therapy Problems (DTPs), more appropriate medication use, reduced healthcare utilization and medication costs, and improved management of chronic diseases.^4-8^ Integration of pharmacists into interprofessional primary care teams has advanced globally over the past decade^9-13^ and is advocated by several Canadian professional organizations.^14-16^ Most recently, both the Canadian Pharmacists Association^17^ and the Canadian Medical Association^18^ have called for a renewed investment in team-based care, not only to leverage its established benefits but also to address urgent challenges with patient access to primary care.^19,20^

In 2018, the government of the province of British Columbia (BC) announced new funding to integrate pharmacists into team-based primary care.^21^ This was part of a large-scale initiative by the BC Ministry of Health to enhance primary care delivery across the province, where healthcare providers collaborate and complement each other within family medical practice settings in geographic areas known as Primary Care Networks (PCNs).^22^ The Pharmacists in PCN Program (the program) was led by the University of British Columbia Faculty of Pharmaceutical Sciences working in collaboration with the BC Ministry of Health, regional health authorities, PCNs, and associated communities. The program integrated Primary Care Clinical Pharmacists (PCCPs) as members of the Interprofessional Team (IPT).^6,23,24^ Pharmacists were expected to offer expertise in drug therapy decision-making for complex patients, reduce the number and severity of DTPs, and reduce unnecessary and negative drug therapy outcomes for patients. This was also expected to increase patient, physician, pharmacist, and IPT members’ satisfaction and increase information sharing and collaboration between pharmacists and other members of the patient care team throughout the PCN community. The program was implemented over 3 years, from October 1, 2020, to September 30, 2023. Pharmacists provided care in 47 PCNs over the implementation period, cared for 7,456 unique patients, and conducted over 24,000 patient appointments. Further details of the program and overall implementation outcomes are published elsewhere.^24^

Experiences of program implementation and pharmacist integration were sought from many perspectives. Unique to our program evaluation was seeking the experiences and perspectives of PCN administrators such as directors, managers, and coordinators who worked closely with the program team and PCCPs over the 3-year implementation period. The objectives of this article are to describe (i) PCN administrators’ perspectives and experiences during the integration of pharmacists into a team-based primary care model in BC and (ii) the facilitators of and barriers to interprofessional care within the evolving team-based care model.

## Methods

### Design, Population, and Participant Recruitment

A survey was deployed over a 6-week period from May 8 to June 16, 2023, with a questionnaire administered using Qualtrics® in English only. The questionnaire was designed to elicit experiences and perspectives from PCN healthcare administrators who collaborated with the program team during program implementation and who supported PCCP integration. The questionnaire was developed by members of the evaluation team following review of the literature and internal discussion to define the domains important to address the objectives of the evaluation. The questionnaire consisted of 27 questions divided into 5 domains (number of questions): (i) about your PCN and role (11); (ii) PCCP referrals and scheduling (2); (iii) experiences working with the UBC program team (3); (iv) experiences working with PCCPs (5); and (v) readiness for adoption of PCCP in your practice (6). Prior to deployment, the questionnaire underwent testing internally at the UBC Faculty of Pharmaceutical Sciences for both content and functionality by both clinicians and staff. Evaluation coordinators also completed testing for functionality within Qualtrics®.

Administrators in all PCNs who worked directly with the program team during implementation were eligible to participate if the PCCP had been integrated in their PCN for ≥6 months. A person-specific link to the questionnaire was sent by email, which began with an informed consent. If the survey was not completed after 2 weeks, an email reminder was sent. A second and final email reminder was sent 1 week prior to the closure of the survey. Participation in the survey was voluntary, and there were no mandatory questions. Questionnaire items included Likert scales, multiple-option questions, and free-text fields.

Incentives were offered to participants who completed the questionnaire and voluntarily disclosed their email address, which included a 1 in 5 chance to win an $80 gift card.

### Data Analysis

Only completed items were analyzed, and response rates were reported per item. Qualitative content analysis of open-ended responses was performed on questions designed to capture participants’ perspectives on facilitators and barriers to pharmacists’ integration and for effective delivery of team-based primary care. De-identified free-text responses were imported into Excel and analyzed. Content analysis involved deriving codes and categories inductively from the data without imposing preconceived categories or frameworks.^25^ Two researchers (PZ/AN) independently performed these analyzes. Iterative review and discussion between PZ and AN were used to reach consensus and themes were refined to best represent the perspectives expressed.

Results are reported using the Checklist for Reporting of Internet E-Surveys (CHERRIES) guidelines.^26^

### Ethics Approval

The study sponsor (BC Ministry of Health) completed a privacy impact assessment, and the University of British Columbia (UBC) Behavioural Research Ethics Board determined that no ethical approval was required for this survey based on Article 2.5 of the Canadian Tri-Council Policy Statement: Ethical Conduct for Research Involving Humans.^27^

## Results

Overall, 81 PCN administrators representing 22 PCN communities (40 PCNs) were invited to participate. Among those invited, 51 (65.4%) responded representing 20 PCN communities (38 PCNs) and were included in the analyzes. Table 1 outlines the demographics of the participants.

**Table 1.** Demographic Characteristics of Respondents (N = 51)

	n	%
Role		
Manager	19	37.4
Executive Director/Director	11	21.6
Lead	10	19.6
Coordinator/Supervisor	6	11.8
Coach	2	3.9
Unspecified	3	5.8
Healthcare professional training		
Yes	24	47.0
No	27	53.0
Primary stakeholder organization		
PCN/Division of Family Practice	32	62.8
Health Authority	19	37.2
Health Authority PCN location		
Island	18	35.3
Fraser	12	23.5
Vancouver Coastal	11	21.6
Interior	9	17.6
Northern	1	2.0
Time in PCN		
>5 years	9	17.7
>2-5 years	24	47.1
1-2 years	12	23.5
<12 months	6	11.8

Respondents report the predominant model of care the PCN uses to connect members of the IPT, including blended (67.4%, 33/49) with less in a fully co-located (18.4%, 9/49) or hub and spoke (14.3%, 7/49) model.

## Experience Working with UBC Program Team

**Figure 1.**
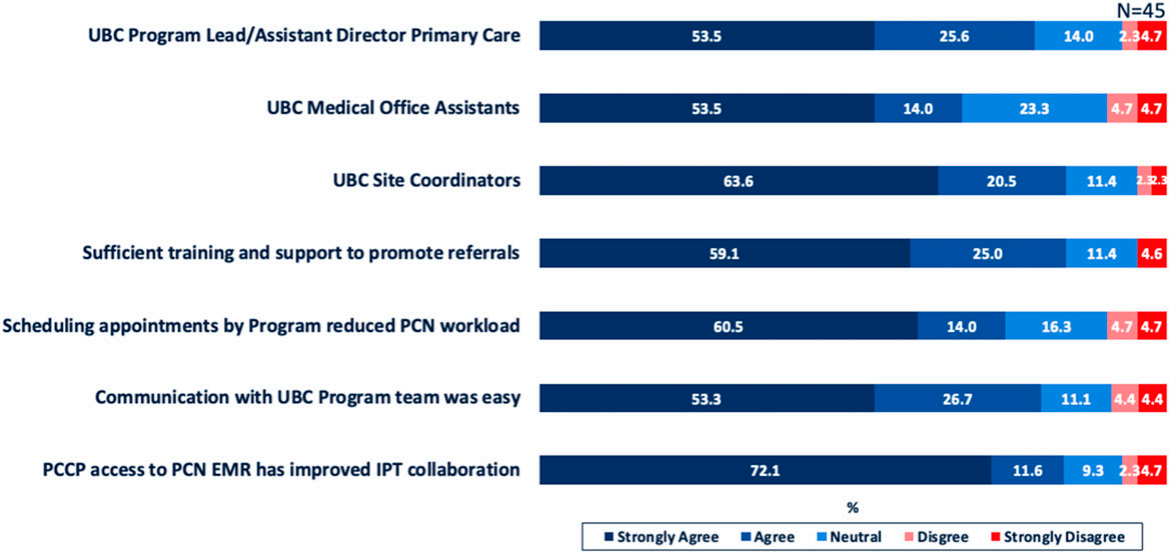
Satisfaction of PCN Administrators with Interactions with UBC Program Team and Related Logistical Support and Communication

Overall, 88.9% (40/45) were satisfied/very satisfied with the experience and effectiveness working with the UBC program team during implementation. Specifically, Figure 1 describes the extent to which members of the UBC program team, and related logistical and communication supports provided, were effective and important with integration of PCCPs into their respective PCNs.

## Experience Working with PCCPs

Overall, 95.6% (43/45) were satisfied/very satisfied with the experience and effectiveness of working with their PCCP, and 95.3% (41/43) felt that more PCNs/healthcare providers would benefit from access to a PCCP. Participants were asked to rate their opinion of the importance of including a PCCP within the PCN to care for complex patients using a 10-point scale (1 = not at all important to 10 = very important). More than 74% (32/43) indicated very important (10/10) while 97.7% (42/43) scored the response in the 8-10 category, suggesting consensus support.

All respondents agreed/strongly agreed that PCCPs were effective members of the IPT (100%, 43/43), added value to team-based care (100%, 44/44), and were capable of helping improve drug therapy outcomes for complex patients (100%, 43/43).

Most respondents agreed/strongly agreed that PCCPs maintained trust and professional relationships with patients (94.9%, 37/39) and IPT members (87.5%, 35/40) through active listening and showing respect of opinions, privacy, and dignity of others. All respondents (100%, 42/42) felt the PCCP was accessible and approachable and also demonstrated professional, clear, and direct verbal (100%, 41/41) and written (100%, 36/36) communication skills. All respondents (100%, 42/42) also felt that the PCCP respected the roles of others and worked constructively with their team. In addition, all respondents (100%, 36/36) felt that PCCPs were effective in handing over work and acted in the best interests of the IPT members and patient care. Finally, 97.6% (40/41) of respondents agreed/strongly agreed that the PCCPs took responsibility for following through on tasks and were responsive to questions or requests they received.

## Integration

Participants were asked to define factors that facilitated and hindered effective pharmacist integration into the PCNs. Facilitators included (number of responses): (i) interprofessional collaboration built on shared goals, trust, respect, and commitment to patient-centred care (21); (ii) co-location of the pharmacist with the patient care team (14); (iii) role clarity and clear scope of practice, facilitated via onboarding and education (14); (iv) efficient communication and coordination with IPT members and administrators (13); (v) enabled and functional PCN EMR access facilitating information sharing between IPT members (13); (vi) clear and flexible systems, processes, and workflows including referral pathways and service access (12); and (vii) resources including administrative support, overhead funding, and supportive leadership (12). Barriers included: (i) lack of pharmacist co-location with all IPT members (14); (ii) EMR challenges with access and integration preventing efficient sharing of documentation (14); (iii) heterogeneity of processes, goals, and resources among team members including stage of PCN development/readiness (12); (iv) limited pharmacist capacity to care for large patient populations or practice across multiple PCN clinics (10); (v) lack of role clarity, scope, value, and awareness and unclear expectations (9); (vi) physician/PCCP/IPT resistance to team-based care or unwillingness to support interprofessional collaboration (8); and (vii) lack of resources such as overhead funding, space, and administrative support (6).

### Facilitators of and Barriers to Team-Based Care

Participants were asked to define their top 3 factors that facilitated and top 3 factors that hindered effective team-based care. Facilitators covered 7 themes, while barriers covered 9 themes outlined in Table 2.

**Table 2.** Facilitators Are Barriers to Team-Based Care (Frequency of Response)

Facilitators	Barriers
• Effective oral and written communication between IPT members facilitated via a shared EMR (32)	• Lack of integrated EMR preventing efficient access and sharing of patient information and other communication (17)
• Interprofessional collaboration build on trust, respect, and commitment to patient-centred care (19)	• Lack of role clarity and scope of practice among team members (14)
• In-person communication and co-location of the patient care team (19)	• Lack of co-location precluding relationship building, timely care, and optimization and efficiency of the team (13)
• IPT members who are approachable, flexible, and accountable (12)	• IPT members not being available, accessible, flexible, or approachable (12)
• Role clarity and clear scope of practice of all IPT members (10)	• Limited capacity of members of team to care for large patient populations or practice among many PCN clinics often over large geographic areas (11)
• IPT members that are accessible and available for both patient care and other team-related activities (8)	• Lack of resources to support the IPT including overhead funding, space, and ability to backfill vacant positions (11)
• Strong support provided to all team members for orientation, onboarding, continued education, and professional development (7)	• Inefficient or absent communication between IPT members (9)
	• Heterogeneity of processes, goals, and resources among team members (6)
	• Challenging and rigid referral systems and requirements (6)

Overall, when asked about how well they felt administrative systems and interprofessional teams were integrated to support patient care within their PCN, 46.3% (20/43) felt that their PCN is somewhat integrated with some evidence of interprofessional collaboration and shared care but no centralized patient referral system. However, 34.9% (15/43) felt that their PCN was almost fully integrated, with established centralized patient referral systems and care provider databases.

## Discussion

Team-based primary care has established benefits for patients, team members, and the healthcare system.^14,15,28,29^ Though primary care pharmacy practice is more established in other Canadian provinces, it was important to understand the BC experience of integrating pharmacists as members of the team in order to support quality improvement, sustainability, and expansion of the program. Our survey of healthcare administrators within the PCN was important to supplement the experiences shared by patients,^30,31^ pharmacists and members of the IPT. Primary care administrators reported overall positive experiences working with the program team during implementation and with the pharmacists within their respective PCNs. The facilitators and barriers to integration of the pharmacist into PCN during implementation offer areas to focus as the program continues to optimize the integration and development into the IPT, including further opportunities for pharmacists to optimize training and skills to support their own integration into team-based primary care.^32^

More broadly than experiences with the pharmacist, administrators were able to offer a lens to their experiences overall in the development of the IPT to support care delivery within their respective PCNs. The facilitators to effective team-based care identified in our survey are intuitive, while barriers offer an opportunity for reflection on how to further enhance the integration and support successful collaboration of all members of the IPT in primary care. Some of the barriers identified reflected the evolving BC primary model during the survey period. As observed in other elements of program evaluation, including perspectives from pharmacists and other members of the IPT, system readiness for team-based care was a challenge, and many of the Canadian College of Family Physicians’ 10 pillars for team-based care within a Patient Medical Home^28^ were not in place or optimized when team members were being integrated, particularly appropriate infrastructure and connected and accessible care. In addition, the approach to team-based care was heterogeneous across the province. Some of these barriers may have been at least partially attributable to insufficient commitment by healthcare administrators, clinicians, or other stakeholders to the concepts and principles of team-based primary care. As such, IPT members in PCNs often struggled working together due to system-level challenges during model development.

In the BC model, pharmacists and other IPT members were often not co-located, instead practicing in a hub-and-spoke model where IPT members were remotely located away from each other or together but physically located away from the family physicians and nurse practitioners. Although this may not be as relevant for other disciplines, in BC, pharmacists have limited prescribing authority, and most medication changes require a physician or nurse practitioner to prescribe them. As such, their ability to optimize medication use for patients required effective communication and relationships with prescribers, which was not always accomplished and further challenged by lack of co-location.

Our findings related to facilitators and barriers to effective team-based care are consistent in many ways with reports from others.^1,2,19,33-35^ Jorgenson et al^33^ described facilitators and barriers that primary care teams experience during pharmacist integration in the province of Saskatchewan included (i) relationships, trust, and respect; (ii) pharmacist role definition; (iii) orientation and support; (iv) pharmacist personality and professional experience; (v) pharmacist presence and visibility; (vi) resources and fundings; and (vii) value of the pharmacist role. Rawlinson et al^35^ report results from a systematic review of 29 studies with the most frequent facilitators being, (i) availability of funding, supportive policies, incentives, and compensations for professionals at the system level; (ii) reorganizing practices and team structure, co-location, tools for care processes, and providing training and sufficient human resources at the organizational level; (iii) the quality of communication, respect and cohesion between professionals, and a shared power at the inter-individual level; and (iv) a positive attitude towards interprofessional care at the individual level. Barriers included (i) lack of long-term funding and inadequate reimbursement policies; (ii) lack of time, leadership at the organizational level, and insufficient training; (iii) lack of clear role boundaries and responsibilities, poor communication, professional identity, and power issues at the inter-individual level; and (iv) doubts regarding the benefits of IPC and resistance to change at the individual level.

One factor that may be unique to the evaluation we undertook is that the model of team-based care in BC was evolving at the time of this survey, and PCNs were at various degrees of development. In addition, the order in which IPT members were integrated was also not uniform, so perspectives related to the temporal relations with the maturing of the PCN development and construction of the team may have influenced experiences described by PCN administrators. Future assessment of identified experiences, facilitators, and barriers is warranted to evaluate changes over time once all IPT members are established and working together in a more developed team-based care model in BC which continues to evolve and expand.

Our survey had several strengths, which included a high response rate (>65%) with a population representative of PCN administrators from across the province. The survey also explored both PCN administrators’ experiences during implementation with the program team and with the pharmacists as well as facilitators of both effective pharmacists integration, but more broadly to effective team-based care, an outcome administrators were charged with being responsible for their respective PCNs, and a unique perspective no other stakeholder could provide with the same degree of detail. This lens from PCN administrators added context to the experiences shared by pharmacists and PCN IPT members in other aspects of program evaluation. Limitations of our survey include a potential for response bias inherent in survey methods. The survey was only distributed in English and to those who gave consent to share their email, thus limiting access and/or participation of some IPT members. Our findings explored PCN administrators’ experiences in a team-based primary care model and may not be generalizable to other models of primary care where pharmacy practice may be different, or to similar yet more established models.

## Conclusion

Health service administrators from PCNs reported an overall high degree of satisfaction with their experiences collaborating with the program team and with pharmacists during implementation of team-based primary care model in BC. Facilitators to successful team-based care should be further supported while identified barriers should be addressed to optimize the effectiveness and efficiency of the entire team as the model of primary care continues to evolve.

## Data Availability

Data generated during and/or analyzed during the current study are not available. However, inquiries regarding data can be directed to the corresponding author.*
